# Influence of Multiple Factors on the Workability and Early Strength Development of Alkali-Activated Fly Ash and Slag-Based Geopolymer-Stabilized Soil

**DOI:** 10.3390/ma15072682

**Published:** 2022-04-06

**Authors:** Xinyu Li, Yufei Zhao, Yong Hu, Guanci Wang, Minmin Xia, Biao Luo, Zhengdong Luo

**Affiliations:** 1 Hunan Xihu Construction Group Co., Ltd., Changsha 410013, China; lixinyu0102@126.com; 2 China Institute of Water Resources and Hydropower Research, Beijing 100038, China; 3 Yueyang City Roads and Bridge Construction Corporation, Yueyang 414021, China; huyong02@sohu.com (Y.H.); wangguanci01@sohu.com (G.W.); 4 Department of Mechanical and Electrical Engineering, Hunan Communication Polytechnic, Changsha 410132, China; 5 College of Civil Engineering and Mechanics, Xiangtan University, Xiangtan 411105, China; luozhengdong0425@163.com

**Keywords:** alkali-activated, fly ash and slag-based geopolymer, stabilized soil, workability, unconfined compression strength

## Abstract

The complexity of composite geopolymer materials results in instability in the setting and hardening of geopolymer-stabilized soil. In order to determine the appropriate mix proportion scheme for composite geopolymer-stabilized soil, this study investigated the effects of two preparation methods, fly ash/slag ratio and alkali activator modulus, on workability and strength development trends in alkali-excited fly ash and slag-based geopolymer-stabilized soil. The results showed that the high ambient temperatures created by the one-step method were more conducive to the setting and hardening of the geopolymer-stabilized soil; its 3 d/28 d UCS (unconfined compression strength) ratio was 62.43–78.60%, and its 7 d/28 d UCS ratio was 70.37–83.63%. With increases of the alkali activator modulus or the proportion of fly ash, the setting time of stabilized soil was gradually prolonged, and its fluidity increased. Meanwhile, the strength development of stabilized soil was significantly affected by the proportion of fly ash and the alkali activator modulus; the maximum UCS value was obtained at II-2-O, prepared by the one-step method, with an alkali activator modulus of 1.2 and a fly ash/slag ratio of 20/80. Specifically, the 3, 7, and 28 d UCS values of II-2-O were 1.65, 1.89, and 2.26 MPa, respectively, and its 3 d/28 d UCS ratio and 7 d/28 d UCS ratio were 73.01% and 83.63%, respectively. These results will be of great importance in further research on (and construction guidance of) composite geopolymer-stabilized soil.

## 1. Introduction

As the main building material in the modern construction industry, the production of cement is accompanied by high emissions and pollution levels [1]. It has been estimated that 550 kg of CO_2_ is produced per 1000 kg of cement produced, and 400 kg of CO_2_ is released into the atmosphere due to fuel combustion [2,3,4]. This accounts for about 5–7% of global carbon dioxide emissions [5,6]. Obviously, the large-scale use of cement invisibly increases the energy consumption and cost of buildings. Looking for green cementitious materials to replace cement has become a hot spot in the field of modern engineering. Geopolymers are a kind of three-dimensional network of inorganic polymers obtained by appropriate processing of aluminosilicate materials and alkali activators. They have similar cementitious properties to cement [7,8,9,10]. Their coagulation and hardening are divided into two processes: depolymerization and polycondensation. In the presence of an alkali activator, the silicon–oxygen bond and aluminum–oxygen bond of aluminosilicate break to form oligomeric silicon-alumina tetrahedron. Then, the oligomeric aluminosilicate uses water as a medium to reassemble into a new Si-O-Al network system [11,12]. At present, raw materials for geopolymers are generally obtained from industrial solid wastes, such as fly ash and blast furnace slag [13,14,15,16]. Compared to cement, the production of geopolymer can reduce energy consumption by about 60% and reduce carbon emissions by 80–90% [17,18,19,20,21]. In addition, the secondary utilization of industrial solid waste is realized, which has a positive impact on environmental protection [22,23,24,25]. As such, geopolymers are considered an ideal substitute for cement and have seen increasing use in various engineering construction practices.

The application of geopolymers as stabilizers in foundation treatment has received extensive attention recently. It is considered an effective way to improve the strength of stabilized soil and reduce costs. Rios et al. [26] proposed the use of low-cost geopolymers prepared from alkali-excited low-calcium fly ash to stabilize silt, and the test results confirmed that such geopolymers were comparable to cement as a foundation treatment material. Cristelo et al. [27] found that the unconfined compressive strength (UCS) of alkali-excited fly ash geopolymer-stabilized soils increased to 900 kPa due to the formation of a large amount of aluminosilicate gel (N-A-S-H). Furthermore, the shear strength of the fly ash-based geopolymer-stabilized soil was also improved [28]. Dassekpo et al. [29] proposed that the weight proportion of soil and fly ash was determined according to the whole process effect of geopolymers on particle interaction. Poor-quality soil treated with a slag-based alkaline paste showed maximum UCS values and was environmentally friendly, compared with the soft soil stabilized by single cement, lime, and fly ash [30,31]. Existing research showed that the stability of soft soil was significantly affected by factors such as slag content, modulus, and dosage of alkali activators [32,33,34]. Further studies found that lower moisture content and composite geopolymer materials were helpful in reducing poor bonding between the geopolymer and soil particles [35,36,37,38]. Luo et al. [39] proposed using slag/fly ash composite as a stabilizer for soft soil; the measured 28-day UCS of stabilized soil was higher than that of undisturbed soil (56.0 times higher) and cement-stabilized soil (1.39 times higher). Singhi et al. [31] found that Si/Al and Na/Al ratios were important factors affecting the soil–geopolymer system (the main raw materials of which were fly ash and slag), and different mixing ratio schemes corresponded to different stabilization effects. At present, the polymerization process, strength development laws, and reaction products of geopolymer-stabilized soils have a certain research basis, but there are still many problems in the geopolymer–soil system that have not been uniformly recognized [40], especially in terms of workability. As an example, the initial setting process of slag-based geopolymer is usually completed within 10–30 min [41], while the hardening time of fly ash-based geopolymer cured at room temperature may exceed 24 h [42]. Instability of setting and hardening is not conducive to the engineering application of geopolymers in foundation treatment. The diversity of raw materials and the variability of alkali activators together create complexity in geopolymer-related research [43].

In addition to the material composition and properties of the composite geopolymer itself, the influence of the preparation method on the workability and mechanical properties of geopolymer-stabilized soil cannot be ignored. In the above studies on geopolymer-solidified soils, most of them adopted a two-step preparation method [44]. Specifically, in the two-step method, the silicon-alumina raw material is first reacted with an alkali activator solution, and then the obtained geopolymer paste is used for soil stabilization. However, this method comes with the disadvantages of difficult transportation and storage of alkali activators, which affects the efficiency of on-site construction. The one-step method refers to directly mixing the silicon-alumina material with the dry alkali activator [45], which reduces the intermediate links in the preparation and transportation of the alkali activator. However, to date, there have been few relevant works in the literature on the effects of preparation methods on the stabilization of fly ash and slag-based geopolymer-stabilized soil. In order to further promote the practical application of composite geopolymer in the treatment of soft soil, this study analyzed the setting time, fluidity and strength development of geopolymer-stabilized soil from various perspectives, such as preparation method, fly ash/slag ratio and alkali activator modulus. The research results could provide an appropriate mix proportion scheme for the application of an alkali-activated fly ash/slag composite system to replace cement in soft soil stabilization, not only effectively alleviating environmental problems related to cement, but also realizing the reduction, resource, and harmless utilization of fly ash and slag.

## 2. Materials and Test Program

### 2.1. Materials

In this study, the soil used was a typical red clay from Changsha, China, taken from a subterranean depth of 6.8 m. The liquid limit and plasticity indices of the soil were 46% and 27%, respectively, and its wet density was 1.47 g/cm^3^. The soil samples were air-dried and ground, then dried in an oven at 80 °C, and finally passed through a 2.36 mm mesh sieve.

The fineness of the Class F fly ash used was 0.015 mm, and its weight of screen residue was 10.7%. The slag used was S95 ground granulated blast furnace slag with a specific surface area of 424 m^2^/kg. Both fly ash and slag were provided by Gongyi Longze Water Purification Material Co., Ltd., Zhengzhou, China. The detailed chemical composition of the above materials, measured according to GB/T 176-2017, is shown in Table 1 [46]. The composite alkali activator was composed of sodium silicate (Na_2_SiO_3_) solution and NaOH in a certain proportion. The chemical equation of the used Na_2_SiO_3_ solution was Na_2_O·*n*SiO_2_, its initial modulus (*n*) was 3.10 (where *n* = *m*(SiO_2_)/*m*(Na_2_O), and the mass fractions of Na_2_O and SiO_2_ were 9.22% and 28.58%, respectively).

### 2.2. Mix Proportions and Sample Preparation

In this study, the reshaped soil was prepared according to the actual moisture content of 48.0%. The stabilizer was taken as 15% of the soil mass, and the alkali activator content was 30%. The water introduced by the Na_2_SiO_3_ solution was taken into account to ensure a constant water/binder ratio (*w*/*b*) of 0.5. The detailed mix proportions are shown in Table 2. Among them, I, II, III in the specimen labels correspond to fly ash/slag ratios (F/S) of 10/90, 20/80, and 30/70, respectively; numbers 1–5 represent the modulus gradient of alkali activator modulus (0.9, 1.2, 1.5, 1.8, 2.1); O/T represents the preparation method (one-step method, represented by O, and two-step method, represented by T).

In this study, an NaOH tablet was used to adjust the modulus of Na_2_SiO_3_ solution; the specific calculation formula was as follows:

(1) $${\mathrm{Na}}_{2}O\cdot n{\mathrm{SiO}}_{2}+m\mathrm{NaOH}\to (1+\frac{m}{2}{)\mathrm{Na}}_{2}O+n{\mathrm{SiO}}_{2}+\frac{m}{2}H_{2}O$$

(2) $$N=\frac{n}{1+\frac{m}{2}}$$

In Equations (1) and (2), *N* is the target modulus of the Na_2_SiO_3_ solution, *n* is the initial modulus of the Na_2_SiO_3_ solution, and *m* is the amount of the required NaOH substance (unit mol).

The one-step method for preparing the mixture was as follows: the fly ash and slag were mixed with NaOH dry powder, and the mixed solution of water and Na_2_SiO_3_ solution was added to prepare a geopolymer, which was used as a stabilizer.

The two-step method preparation procedure was as follows: a certain mass of fly ash and slag were weighed and mixed uniformly in the stirring pot, and the cooled alkali activator solution was added. First, it was stirred at 140 r/min for 3–5 min, then adjusted to 280 r/min and stirred for 1–2 additional min. Finally, it was stirred at a rate of 280 r/min for 2–3 min until the geopolymer stabilizer and the reshaped soil were uniformly mixed.

After completing the mixing procedure as described above, fluidity and setting time tests were performed. Meanwhile, the mixture was sequentially filled in three layers into a 70.7 × 70.7 × 70.7 mm cube mold. After each layer was filled, it was placed on a vibrating table and vibrated evenly to remove air bubbles in the specimen until the molding was completed. The specimens were covered with plastic wrap and cured at room temperature for 1 d, and then demolded. The exposed specimens were re-covered with plastic wrap and cured until the target age in a standard environment (temperature of 20 ± 2 °C, RH of 95 ± 3%).

### 2.3. Test Methods

#### 2.3.1. Setting Time

The setting time of the fresh mixture was determined using the Vicat apparatus mold. The judgment basis for when the mixture had reached the initial setting state was when the maximum sinking depth of the initial setting test needle reached 4 ± 1 mm from the bottom plate. The mixture was immediately judged to have reached the final setting state when the annular attachment of the final setting test needle was unable to leave a trace on the sample or when the sinking depth was less than 0.5 mm [47].

#### 2.3.2. Fluidity

The NLD-3 cement mortar fluidity tester was used to measure the diffusion diameter of the free-flowing mixture on the flow plate. The fluidity value of the mixture was taken from the average value of two mutually perpendicular diameters [48].

#### 2.3.3. Unconfined Compressive Strength (UCS)

**Figure 1:**
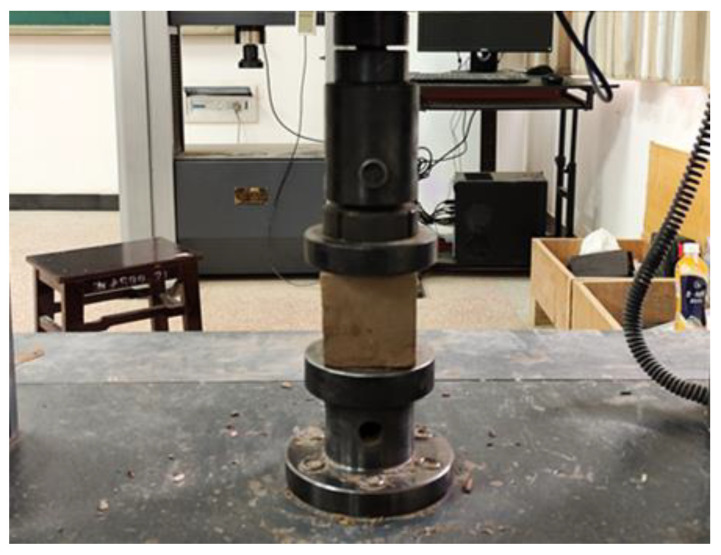
UCS test.

According to the experimental method in [39], the 3, 7, and 28 d UCS values of the stabilized soil specimens were tested by a universal testing machine, as shown in Figure 1.

## 3. Results and Discussion

### 3.1. Setting Time

**Figure 2:**
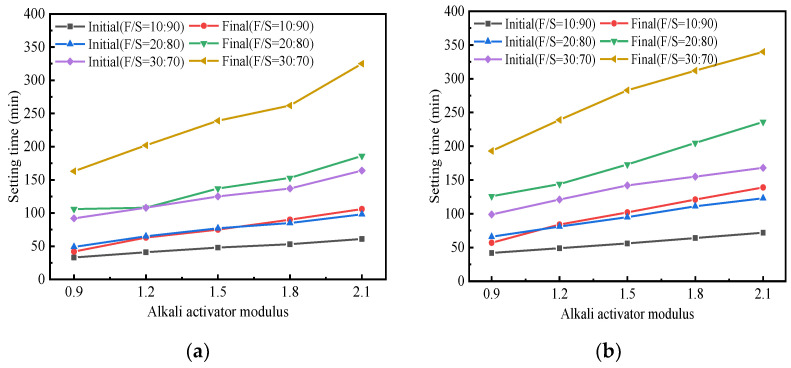
Setting time of stabilized soil: (**a**) One-step method. (**b**) Two-step method.

Figure 2 shows variations in the setting time of geopolymer-stabilized soil under different conditions. The main influencing factors were the preparation method, alkali activator modulus and fly ash/slag ratio (F/S). As observed, the variation trend of the setting time of geopolymer-stabilized soil was roughly the same under different preparation methods. The initial and final setting times of the I-1-O specimen prepared by one-step method were 33 and 42 min, respectively; As for the -1-T specimen prepared by the two-step method, its initial and final setting times were 42 and 57 min, respectively. In contrast, the hardened body of the stabilized soil prepared by the one-step method formed more rapidly, which was attributed to the exothermic heat generated by the contact of NaOH with water. The solubility of aluminosilicate increases with increases in ambient temperature, and more Si and Al active monomers dissolve out and participate in geopolymerization. This is beneficial as it accelerates the formation of the aluminosilicate gel and aluminosilicate core [49]. However, an excessively fast setting and hardening rate of stabilized soil will impair the construction quality.

As shown in Figure 2, in the one-step method, the modulus of the NaOH-modified Na_2_SiO_3_ solution was also one of the important factors affecting the setting behavior of geopolymer-stabilized soil [50,51]. The specific phenomenon was that the setting time of stabilized soil increased gradually with the alkali activator modulus. This was because the activator with higher modulus had a higher viscosity which reduced the migration rate of alkali metal cations and weakened interactions between the reacting species, thereby decreasing the geopolymerization rate and prolonging the setting and hardening time [52].

Under the condition of the same activator modulus, the setting time of geopolymer-stabilized soil increased with the proportion of fly ash (Figure 2a). As an example, the initial setting times of I-2-O, II-2-O and III-2-O were 41, 65, and 108 min, respectively, and the differences between their initial and final setting times were 22, 43, and 94 min, respectively. This was because the vitreous structure on the surface of the fly ash was relatively dense, which increased the difficulty of its excitation, thereby slowing down the speed of setting and hardening of the stabilized soil. Thus, the setting time of stabilized soil can be adjusted by changing the F/S value to fall within a suitable range.

### 3.2. Fluidity

**Figure 3:**
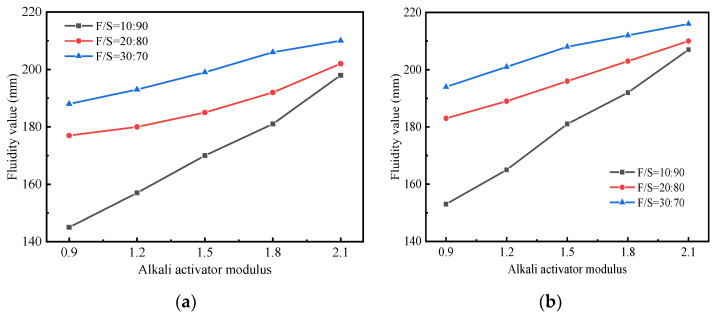
Fluidity of stabilized soil: (**a**) One-step method. (**b**) Two-step method.

The relationship between the fluidity of the stabilized soil and the preparation methods, i.e., alkali activator modulus and fly ash/slag ratio, is shown in Figure 3. As observed, the fluidity values of the stabilized soil samples prepared by the one-step method were consistently lower than those prepared by the two-step method, and the fluidity values decreased with the decrease of the alkali activator modulus. This was attributed to early geopolymerization in the stabilized soil. As the alkali activator modulus gradually decreased, the fluidity values decreased from 198 mm for I-5-O to 145 mm for I-1-O. Furthermore, the F/S value was also one of the influencing factors on fluidity. When the proportion of fly ash reached 30%, the fluidity of III-1-O was 43 mm higher than that of I-1-O. This indicated that the spherical vitreous structure of fly ash directly increased the workability of the stabilized soil, and a higher proportion of fly ash delayed the formation of early geopolymer gels.

### 3.3. Strength Development

UCS values represent an important basis for evaluating the setting and hardening of geopolymer-stabilized soil under different conditions. Table 3 shows the average values of 3/7/28 d UCS for stabilized soils.

#### 3.3.1. Effects of the Preparation Methods on the UCS of Stabilized Soil

**Figure 4:**
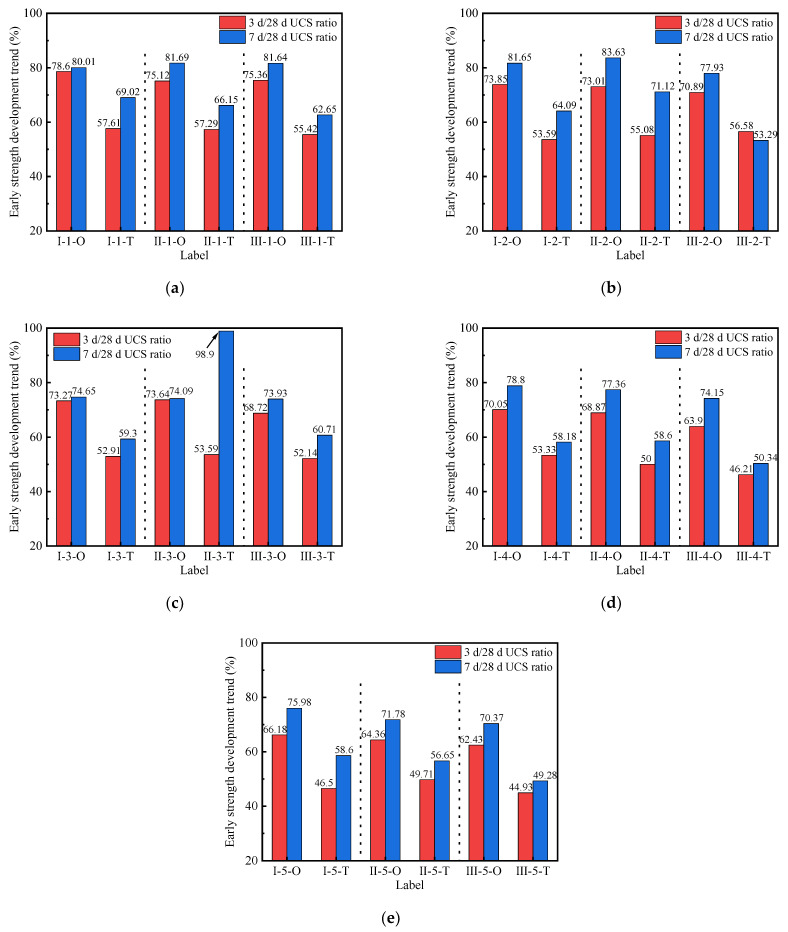
UCS value of stabilized soil with different alkali activator modulus: (**a**) 0.9; (**b**) 1.2; (**c**) 1.5; (**d**) 1.8; (**e**) 2.1.

Figure 4 shows the UCS development trend of geopolymer-stabilized soils under different preparation methods. It can be seen from Figure 4a–e that the early strength development rate of the stabilized soil prepared by the one-step method was significantly higher than in those prepared by the two-step method. In the O series, the 3 d/28 d UCS ratios of stabilized soil were 62.43–78.60%, and the 7 d/28 d UCS ratios were 70.37–83.63%. For the T series, the 3 d/28 d UCS ratios of stabilized soil were 44.93–57.61%, and the 7 d/28 d UCS ratios were 49.27–71.12% (excluding individual values). This indicated that the early strength of the geopolymer-stabilized soil prepared by the one-step method developed relatively fast, and also obtained a higher later strength. This was because the higher ambient temperature facilitated geopolymerization, which in turn increased the compaction of the stabilized soil. At the same time, it was found that a lower alkali activator modulus and lower F/S value also acted synergistically, along with high ambient temperatures, on the strength development of the stabilized soil. Their corresponding specimens had better mechanical properties. Additionally, the 3 d/28 d UCS ratio of II-3-T was 53.59%, while the 7 d/28 d UCS ratio reached 98.90%. The later strength of II-3-T showed almost no increase and was lower than that of the adjacent groups, II-1-T and II-2-T. This showed that the UCS development of stabilized soil prepared by the two-step method was unstable.

#### 3.3.2. Effect of the Fly Ash/Slag Ratio on the UCS of Stabilized Soil

**Figure 5:**
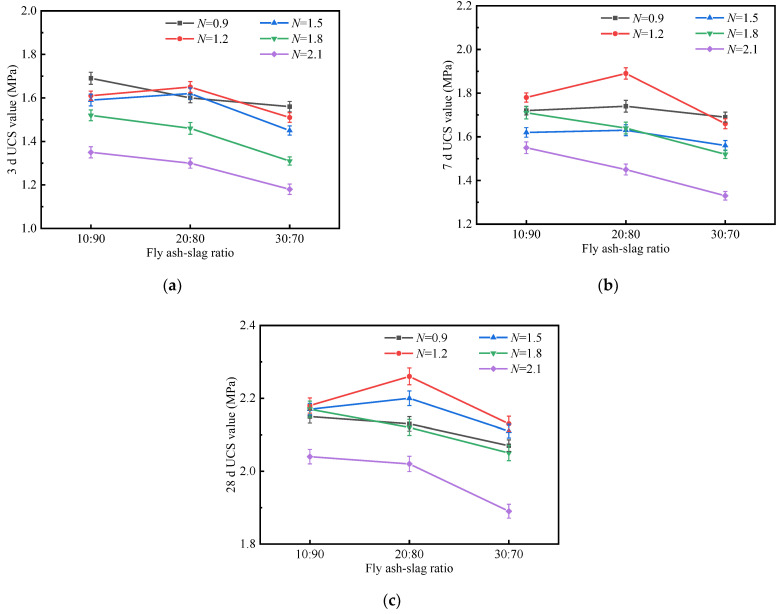
UCS value of stabilized soil with different fly ash/slag ratios: (**a**) 3 d; (**b**) 7 d; (**c**) 28 d.

In view of the fact that the strength development of the fly ash and slag-based geopolymer-stabilized soil prepared by the one-step method was more prominent and stable, we mainly analyzed the test results of the O series in this section. Figure 5 shows the UCS diagrams of stabilized soils with different F/S values.

As illustrated in Figure 5, when the alkali activator modulus was 0.9–1.2, the UCS of the stabilized soil generally increased first and then decreased with the increase of the F/S value. The maximum value was obtained when the F/S value was 20/80. The 3, 7, and 28 d strengths of II-1-O were 1.60, 1.74, and 2.13 MPa, respectively, and the 3 d/28 d UCS ratio and 7 d/28 d UCS ratio of II-1-O were 75.12% and 81.69%, respectively. The 3, 7, and 28 d strengths of II-2-O were 1.65, 1.89, and 2.26 MPa, respectively, and its 3 d/28 d UCS ratio and 7 d/28 d UCS ratio were 73.01% and 83.63%, respectively. In contrast, the early strength growth rate of II-2-O was higher. This indicated that the alkali-excited fly ash/slag system in the stabilized soil reached a suitable state, and the higher strength was attributed to the complex synergistic relationship between the geopolymerization and the CaSiO_3_ hydration reaction [53]. When the alkali activator modulus was 1.5–2.1, the UCS of stabilized soil at each age decreased gradually with the increase of F/S values. Among them, the 28 d UCS of I-5-O, II-5-O and III-5-O were 2.04, 2.02, and 1.89 MPa, respectively; their 3 d/28 d strength ratios were 66.18%, 64.36%, and 62.43%, respectively. Obviously, the increase in the proportion of fly ash not only decreased the later strength, but also delayed the early strength development of the stabilized soil.

#### 3.3.3. Effect of the Alkali Activator Modulus on the UCS of Stabilized Soil

**Figure 6:**
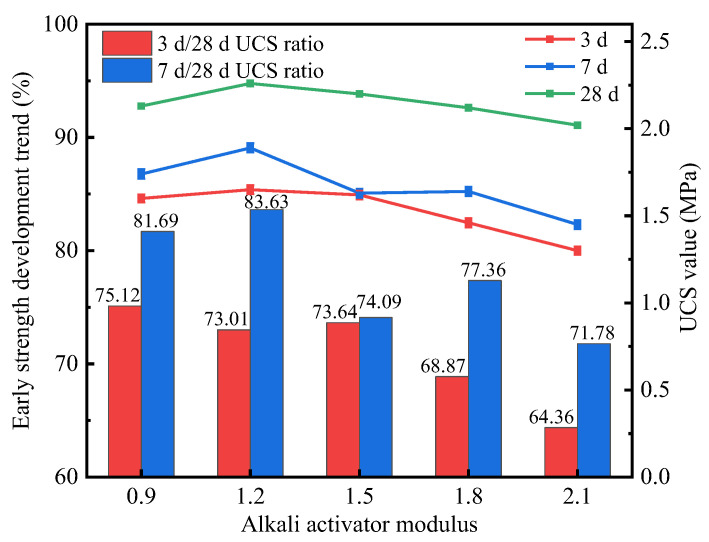
UCS values of stabilized soils with different alkali activator moduli.

The main function of the alkali activator solution was to excite the pozzolanic activities of fly ash and slag, which were more pronounced at high ambient temperatures [54]. As described in Section 3.3.1, the average proportion of 7 d UCS to 28 d UCS of O series stabilized soils reached 70.37–83.63%. Additionally, as observed in Section 3.3.2, the largest 28 d UCS was obtained at a F/S value of 20/80. In view of this, the strength test results of stabilized soil with an F/S value of 20/80 prepared by the one-step method were used as the representative for analysis. Figure 6 shows the strength development trends of II-1-O, II-2-O, II-3-O, II-4-O and II-5-O.

As shown in Figure 6, the strength of the stabilized soil increased when the alkali activator modulus was increased from 0.9 to 1.2, under the conditions that the preparation method was the one-step method and the F/S value was 20/80. In contrast, the 3, 7, and 28 d UCS values of II-2-O were 3.1%, 8.6%, and 6.1% higher than that of II-1-O, respectively. When the alkali activator modulus continued to increase, the strength of the stabilized soil showed a downward trend. Overall, this indicated that 1.2 was the optimal alkali activator modulus for II-2-O with an F/S value of 20/80, representing a good balance between aluminosilicate and alkali activator solutions.

The alkali-excited reactions of geopolymers are mainly a process of synthesizing gel products from substances rich in silicon and aluminum elements under the excitation conditions of alkaline solutions. The alkali activator plays an important role in geopolymerization, providing both hydroxide ions (OH^−^) and sodium ions (Na^+^). Among them, OH^−^ promotes the hydrolysis of the aluminosilicate in the first stage, as presented in Equations (3)–(5) [55], while Na^+^ contributes to the charge balance of the aluminosilicate network.

(3) $${\mathrm{Al}}_{2}O_{3}+3H_{2}O+2{\mathrm{OH}}^{-}\to 2{{\left[\mathrm{Al}(\mathrm{OH}\right)}_{4}]}^{-}$$

(4) $${\mathrm{SiO}}_{2}+H_{2}O+{\mathrm{OH}}^{-}\to {{\left[\mathrm{SiO}(\mathrm{OH}\right)}_{3}]}^{-}$$

(5) $${\mathrm{SiO}}_{2}+2{\mathrm{OH}}^{-}\to {{[\mathrm{SiO}}_{2}{\left(\mathrm{OH}\right)}_{2}]}^{-}$$

## 4. Conclusions

Based on the needs of practical engineering applications, this study focused on the effects of three factors, including preparation method, alkali activator modulus, and fly ash/slag ratio, on the workability and strength development of geopolymer-stabilized soil. The measured parameters included initial and final setting times, fluidity values and UCS (unconfined compressive strength). The following valuable conclusions were drawn:

(1). The development trend of the setting time of the fly ash and slag-based geopolymer-stabilized soil was identical to the fluidity. The setting time and fluidity values of the stabilized soils prepared by the one-step method were slightly lower than those prepared by the two-step method. With increases in of the modulus of the alkali activator or the proportion of fly ash, the setting time of stabilized soil was gradually prolonged, and the fluidity increased.
(2). The one-step method improved the early strength development rate and UCS value stability of the geopolymer-stabilized soil. The 3 d/28 d UCS ratios of the stabilized soil prepared by the one-step method were 62.43–78.60%, and the 7 d/28 d UCS ratios were 70.37–83.63%. As for the stabilized soil prepared by the two-step method, the 3 d/28 d UCS ratios were 44.93–57.61%, and the 7 d/28 d UCS ratios were 49.27–71.12%.
(3). By using the one-step method, the UCS value of stabilized soil first increased and then decreased with the increase of the fly ash/slag ratio when the alkali activator modulus was 0.9–1.2, while the UCS of stabilized soil at each age gradually decreased with the increase of fly ash/slag ratio when the alkali activator modulus was 1.5–2.1.

In this study, a geopolymer-stabilized soil (II-2-O) with an alkali activator modulus of 1.2 and a fly ash/slag ratio of 20/80 was prepared by the one-step method, achieving a good balance between aluminosilicate and alkali activator solution. The 3, 7, and 28 d UCS values of II-2-O were 1.65, 1.89, and 2.26 MPa, respectively, and its 3 d/28 d UCS ratio and 7 d/28 d UCS ratio were 73.01% and 83.63%, respectively.

## Figures and Tables

**Table 1:** Chemical composition of raw materials (wt%).

Raw Materials	SiO_2_	Al_2_O_3_	Fe_2_O_3_	MgO	CaO	SO_3_	Others	LOI
Fly ash	49.04	27.40	1.53	0.86	3.23	1.15	16.79	2.36
Slag	34.50	17.70	1.03	6.01	34.00	1.64	5.12	1.83

**Table 2:** Mix proportions.

Label	Stabilizer Content/%	Alkali Activator		Fly Ash/Slag (F/S)	*w*/*b*
		Modulus (*N*)	Content/%		
I-1-O/T	15	0.9	30	10/90	0.5
I-2-O/T	15	1.2	30	10/90	0.5
I-3-O/T	15	1.5	30	10/90	0.5
I-4-O/T	15	1.8	30	10/90	0.5
I-5-O/T	15	2.1	30	10/90	0.5
II-1-O/T	15	0.9	30	20/80	0.5
II-2-O/T	15	1.2	30	20/80	0.5
II-3-O/T	15	1.5	30	20/80	0.5
II-4-O/T	15	1.8	30	20/80	0.5
II-5-O/T	15	2.1	30	20/80	0.5
III-1-O/T	15	0.9	30	30/70	0.5
III-2-O/T	15	1.2	30	30/70	0.5
III-3-O/T	15	1.5	30	30/70	0.5
III-4-O/T	15	1.8	30	30/70	0.5
III-5-O/T	15	2.1	30	30/70	0.5

**Table 3:** 3, 7, 28 d UCS values of geopolymer-stabilized soils.

Label	Alkali Activator Modulus (*N*)	Fly Ash/Slag (F/S)	UCS Value/MPa (One-Step Method)			UCS Value/MPa (Two-Step Method)		
			3 d	7 d	28 d	3 d	7 d	28 d
I-1-O/T	0.9	10/90	1.69	1.72	2.15	1.06	1.27	1.84
I-2-O/T	1.2	10/90	1.61	1.78	2.18	0.97	1.16	1.81
I-3-O/T	1.5	10/90	1.59	1.62	2.17	0.91	1.02	1.72
I-4-O/T	1.8	10/90	1.52	1.71	2.17	0.88	0.96	1.65
I-5-O/T	2.1	10/90	1.35	1.55	2.04	0.73	0.92	1.57
II-1-O/T	0.9	20/80	1.6	1.74	2.13	1.10	1.27	1.92
II-2-O/T	1.2	20/80	1.65	1.89	2.26	1.03	1.33	1.87
II-3-O/T	1.5	20/80	1.62	1.63	2.20	0.97	1.79	1.81
II-4-O/T	1.8	20/80	1.46	1.64	2.12	0.93	1.09	1.86
II-5-O/T	2.1	20/80	1.30	1.45	2.02	0.86	0.98	1.73
III-1-O/T	0.9	30/70	1.56	1.69	2.07	0.92	1.04	1.66
III-2-O/T	1.2	30/70	1.51	1.66	2.13	0.86	0.81	1.52
III-3-O/T	1.5	30/70	1.45	1.56	2.11	0.73	0.85	1.40
III-4-O/T	1.8	30/70	1.31	1.52	2.05	0.67	0.73	1.45
III-5-O/T	2.1	30/70	1.18	1.33	1.89	0.62	0.68	1.38

## Data Availability

Not applicable.
